# Evaluating the effect of sample type on American alligator (*Alligator mississippiensis*) analyte values in a point-of-care blood analyser

**DOI:** 10.1093/conphys/cov065

**Published:** 2016-01-08

**Authors:** Matthew T. Hamilton, John W. Finger, Megan E. Winzeler, Tracey D. Tuberville

**Affiliations:** 1 Savannah River Ecology Laboratory, University of Georgia, Aiken, SC 29802, USA; 2 Warnell School of Forestry and Natural Resources, University of Georgia, Athens, GA 30602, USA; 3 Department of Biological Sciences, Auburn University, Auburn, AL 36849, USA

**Keywords:** American alligator, biochemistry, i-STAT, lactate, plasma, point-of-care

## Abstract

The assessment of wildlife health has been enhanced by the ability of point-of-care (POC) blood analysers to provide biochemical analyses of non-domesticated animals in the field. However, environmental limitations (e.g. temperature, atmospheric humidity and rain) and lack of reference values may inhibit researchers from using such a device with certain wildlife species. Evaluating the use of alternative sample types, such as plasma, in a POC device may afford researchers the opportunity to delay sample analysis and the ability to use banked samples. In this study, we examined fresh whole blood, fresh plasma and frozen plasma (sample type) pH, partial pressure of carbon dioxide (*P*CO_2_), bicarbonate (HCO_3_^−^), total carbon dioxide (*T*CO_2_), base excess (BE), partial pressure of oxygen (*P*O_2_), oxygen saturation (sO_2_) and lactate concentrations in 23 juvenile American alligators (*Alligator mississippiensis*) using an i-STAT CG4+ cartridge. Our results indicate that sample type had no effect on lactate concentration values (*F*_2,65 _= 0.37, *P* = 0.963), suggesting that the i-STAT analyser can be used reliably to quantify lactate concentrations in fresh and frozen plasma samples. In contrast, the other seven blood parameters measured by the CG4+ cartridge were significantly affected by sample type. Lastly, we were able to collect blood samples from all alligators within 2 min of capture to establish preliminary reference ranges for juvenile alligators based on values obtained using fresh whole blood.

## Abstract (precis)

Point-of-care blood analyzers provide researchers with the opportunity to collect blood biochemistry data for a variety of non-domesticed organisms while in the field. We investigated the use of an i-STAT analyzer and CG4+ cartridge to analyze whole blood, fresh plasma, and previously frozen plasma samples collected from American alligators.

## Introduction

The management and conservation of wildlife often requires the use of several tools to assess the health and physiological status of organisms in the field (Wikelski and Cooke, 2006; Johnstone *et al.*, 2012). Included in this tool set are a growing number of point-of-care (POC) analysers that are being adapted from human health-care settings to investigate the health of non-domesticated and domesticated vertebrates (Atkins *et al.*, 2010; Stoot *et al.*, 2014). Point-of-care analysers provide researchers with the opportunity to conduct a broad array of biochemical analyses to complement physical examination. Additionally, POC analysers deliver prompt results, require low sample volumes and include an assortment of quality control measures for consistent results (Harrenstien *et al.*, 2005; Atkins *et al.*, 2010), making these analysers an attractive alternative to submitting samples for analysis at a diagnostic laboratory, particularly when working in a remote setting.

The i-STAT hand-held analyser is one of the most widely used hand-held blood analysers, having been used in at least 42 studies involving POC devices and non-domesticated vertebrate species (Stoot *et al.*, 2014). It has the capability and versatility to analyse a diverse array of blood parameters at one time through the use of different disposable cartridges (*VetScan i-STAT 1: Operator*'*s Manual*, 2009). Despite the potential utility of the i-STAT analyser and other POC devices, they suffer from a variety of limitations when used in field settings. Environmental conditions, such as temperature, atmospheric humidity and rain, may limit the functionality and reliability of the POC unit (Stoot *et al.*, 2014). Furthermore, POC devices often rely on species-specific correction values that could influence analysis and subsequent results (Harms *et al.*, 2003; Stoot *et al.*, 2014). As such, there is a need for studies focused on non-domesticated vertebrates (Lewbart *et al.*, 2014) and alternative sample types (i.e. plasma or frozen plasma) to confirm the reliability and repeatability of POC devices in analysing samples from non-domesticated organisms.

The American alligator (*Alligator mississippiensis*) is a long-lived predator that is not only of economic importance in the southeastern USA, but also an indicator of wetland health (Milnes and Guillette, 2008). The American alligator is also the most studied crocodilian species (Ryberg *et al.*, 2002), making it a model organism for crocodilian research. However, limited information has been published with regard to the use of POC devices in crocodilians (Campbell *et al.*, 2010; Olsson and Phalen, 2013). Crocodilian habitat conditions do not facilitate the use of most POC devices in the field (e.g. high humidity and temperatures or exposure to water), which can inhibit the analysis of samples directly after collection (Finger *et al.*, 2015b). This often necessitates the use of alternative sample types, such as frozen plasma, and/or requires reference values for comparing blood and plasma parameters.

In this study, we examined juvenile American alligator pH, partial pressure of carbon dioxide (*P*CO_2_), bicarbonate (HCO_3_^−^), total carbon dioxide (*T*CO_2_), base excess (BE), partial pressure of oxygen (*P*O_2_), oxygen saturation (sO_2_) and lactate concentrations in fresh whole blood, fresh plasma and frozen plasma samples using the i-STAT CG4+ cartridge. Our main objective for this study was to assess concordance in acid–base and blood gas analyte values among different sample types (i.e. whole blood, fresh plasma and frozen plasma). Secondly, we wanted to provide preliminary reference values for juvenile American alligators using a common and widely used POC device. We chose to evaluate this technique using a CG4+ cartridge as part of a broader goal to gain a better understanding of the physiological stress response of American alligators. The capture and restraint of crocodilians frequently causes the animal to thrash and exert intense muscular activity, subsequently causing an acid–base disturbance and a release of lactic acid (Coulson and Hernandez, 1983; Franklin *et al.*, 2003; Olsson and Phalen, 2013). In this study, we captured juvenile American alligators quickly to minimize the effects of capture stress on analyte values in baseline blood and plasma samples.

## Materials and methods

### Alligators and husbandry

Finger *et al*. (2015b) have previously described American alligator enclosure conditions and husbandry protocols for the animals in our study. In June 2013, we obtained 23 juvenile American alligators (2–3 years old) from Rockefeller Wildlife Refuge in Grand Chenier, LA, USA and transported them to a climate-controlled (22.7°C) aquatic animal facility at the Savannah River Ecology Laboratory in Aiken, SC, USA, where we randomly assigned each alligator to one of three concrete stalls as part of another study. Water temperature fluctuated with ambient environmental conditions and was continuously filtered at a depth of 36.58 cm in each stall. We provided alligators with one main basking platform per stall and multiple smaller concrete platforms. Light–dark cycles were controlled by light filtration through fibreglass panes and windows on the top and sides of the building. All experimental protocols were approved by the University of Georgia's Institutional Animal Care and Use Committee (approval number A2014 01-030-Y1-A3).

### Blood collection and sample preparation

We collected whole blood samples from eight juvenile alligators during a 2 h sampling period on 20 September 2014 (Session 1). On 29 November 2014 (Session 2), we collected samples from an additional 15 alligators during a 3.5 h sampling session starting at the same time as Session 1 (12.00 h). Alligators were fed until satiation 1 day before each sampling session. On each sampling day, we collected 1.5 ml blood from the occipital sinus within 2 min of capture using a 25-gauge, 2.54 cm non-heparinized needle and 3 ml syringe (Finger *et al.*, 2015a). We immediately transferred blood samples to a 1.3 ml lithium heparin tube (Becton Dickson, San Antonio, TX, USA) and standardized mixing of the sample by gently inverting the tube three times. We then transferred 95 µl of whole blood to a CG4+ cartridge (Abbot Point-of-Care Inc., Princeton, NJ, USA) for analysis on a VetScan i-STAT 1 POC analyser (Abaxis Inc., Union City, CA, USA) and centrifuged (Cole-Parmer, Vernon Hills, IL, USA) the remaining whole blood sample for 3 min at 1640 *g*. After we had separated the whole blood sample, we aliquoted 95 µl of plasma into an additional CG4+ cartridge and analysed it immediately using the i-STAT portable analyser. We then aliquoted 200 µl of plasma into a 1.5 ml tube and placed the tube on ice for 20 min (standardized for each tube) until being stored at −60°C for later analysis. Following sample collection and handling, we measured the juvenile alligator head length (HL), total length (TL) and cloacal temperature. Finally, we determined the sex of all alligators using blunt-nosed tweezers (Allsteadt and Lang, 1995).

To determine the effects of long-term storage on analyte values, we analysed frozen plasma samples from Session 1 and Session 2 at 31 and 42 days, respectfully, after sample collection. We thawed plasma samples at room temperature for ∼15 min prior to being analysed. Once thawed, we vortexed samples for 5 s before transferring 95 μl into a CG4+ cartridge for analysis.

### Blood analyte analysis

We used an i-STAT hand-held analyser and CG4+ cartridges to obtain analyte values for pH, *P*CO_2_, HCO_3_^−^, *T*CO_2_, BE, *P*O_2_, sO_2_ and lactate from 23 alligator blood and plasma samples (HCO_3_^−^, BE, sO_2_ and *T*CO_2_ were calculated by the analyser). Given that some analytes, such as *P*O_2_, *P*CO_2_ and pH, are temperature dependent (Glass *et al.*, 1985), we measured alligator cloacal temperatures at the time of sample collection to correct for temperature variation among individuals and used temperature-corrected values for subsequent statistical analyses.

### Statistical analysis

We tested assumptions of analysis of variance (ANOVA) with Shapiro–Wilk and Bartlett's tests. When assumptions were violated, we log-transformed data to improve normality. In some instances, log-transformed data still violated these assumptions. Given that ANOVA is fairly robust to violations of normality when sample sizes are large (Glass *et al.*, 1972; Schmider *et al.*, 2010), we proceeded with a repeated-measures ANOVA followed by a pairwise *t*-test with Bonferroni correction. Sample type (i.e. whole blood, fresh plasma and frozen plasma) was used as a factor in the analysis. Dependent variables included all blood analytes. Lactate concentrations in some samples (four whole blood, three fresh plasma and five frozen plasma; Table 1) were below the detectable limit of the analyser and were replaced with zeros before being analysed. We performed linear regressions to examine the relationship between whole blood analytes and sample collection time. We conducted all statistical analyses in R (Version 3.1.0; R Development Core Team, 2014) and accepted significance at α = 0.05. Raw analyte values are presented as means ± 1 SD. Temperature and morphometric measurements are reported as means ± 1 SEM.

**Table 1:** Whole blood, fresh plasma and frozen plasma biochemistry values for captive juvenile American alligators (*Alligator mississippiensis*)

	Whole blood		Fresh plasma		Frozen plasma		
Parameter (abbreviation; unit)	*n*	Range (mean ± SD)	*n*	Range (mean ± SD)	*n*	Range (mean ± SD)	i-STAT reportable range
pH	23	7.48–7.86	23	7.38–7.99	23	7.92–8.20	6.50–8.20
		(7.63 ± 0.11)		(7.73 ± 0.14)		(8.03 ± 0.08)	
Partial pressure of carbon dioxide (*P*CO_2_; mmHg)	23	9.2–25.29	23	7.3–20.06	23	6.35–10.53	5–130
		(17.8 ± 5.41)		(12.70 ± 3.85)		(8.48 ± 1.19)	
Partial pressure of oxygen (*P*O_2_; mmHg)	23	12.87–67.68	23	29.55–51.03	23	104.99–156.9	5–800
		(27.53 ± 14.76)		(39.08 ± 6.23)		(127.00 ± 14.87)	
Base excess (BE; mmol/l)	23	−9.00 to 0.00	23	**−19.00 to 2.00**	23	0.00–13.00	−30 to +30
		(−3.70 ± 2.44)		**(−3.39 ± 4.13)**		(5.17 ± 3.41)	
Bicarbonate (HCO_3_^−^; mmol/l)	23	14.90–26.10	23	**8.30–24.90**	23	18.90–29.60	1–85.0
		(21.24 ± 2.77)		**(19.70 ± 3.54)**		(24.13 ± 2.68)	
Total carbon dioxide (*T*CO_2_; mmol/l)	23	15.00–28.00	23	**9.00–26.00**	23	**19.00–30.00**	5–50
		(22.35 ± 3.07)		**(20.39 ± 3.65)**		**(24.65 ± 2.74)**	
Oxygen saturation (sO_2_; %)	23	72.00–99.00	23	96.00–99.00	23	100.00–100.00	0–100
		(90.83 ± 7.35)		(98.13 ± 0.87)^b^		(100 ± 0.00)^b^	
Lactate (Lac; mmol/l)	19^a^	0.34–4.88	20^a^	**0.30–5.10**	18^a^	**0.73–5.04**	0.30–20.00
		(1.90 ± 1.31)		**(1.92 ± 1.36)**		**(2.07 ± 1.33)**	

Values in bold are statistically not significantly different from those for whole blood samples at α = 0.05 using a repeated-measures ANOVA and a *post hoc* pairwise *t*-test. Values below the detectable range of the analyser were replaced with zeros before analysis, but are not represented in the analyte concentration ranges (mean ± SD) for each sample type. Reportable ranges from the i-STAT analyser were provided by Abbott Point of Care Inc. ^a^Some lactate values were lower than the detectable range of the analyser (<0.30 mmol/l). ^b^No significant difference in analyte values between sample types.

## Results

Mean (±SEM) cloacal temperature at the time of sample collection was 20.73 ± 0.42°C, head length averaged 13 ± 0.13 cm, and mean total length was 104.10 ± 1.10 cm for all alligators (*n* = 23; 19 female and four male) included in this study.

### Effect of sample type on analytes

**Figure 1:**
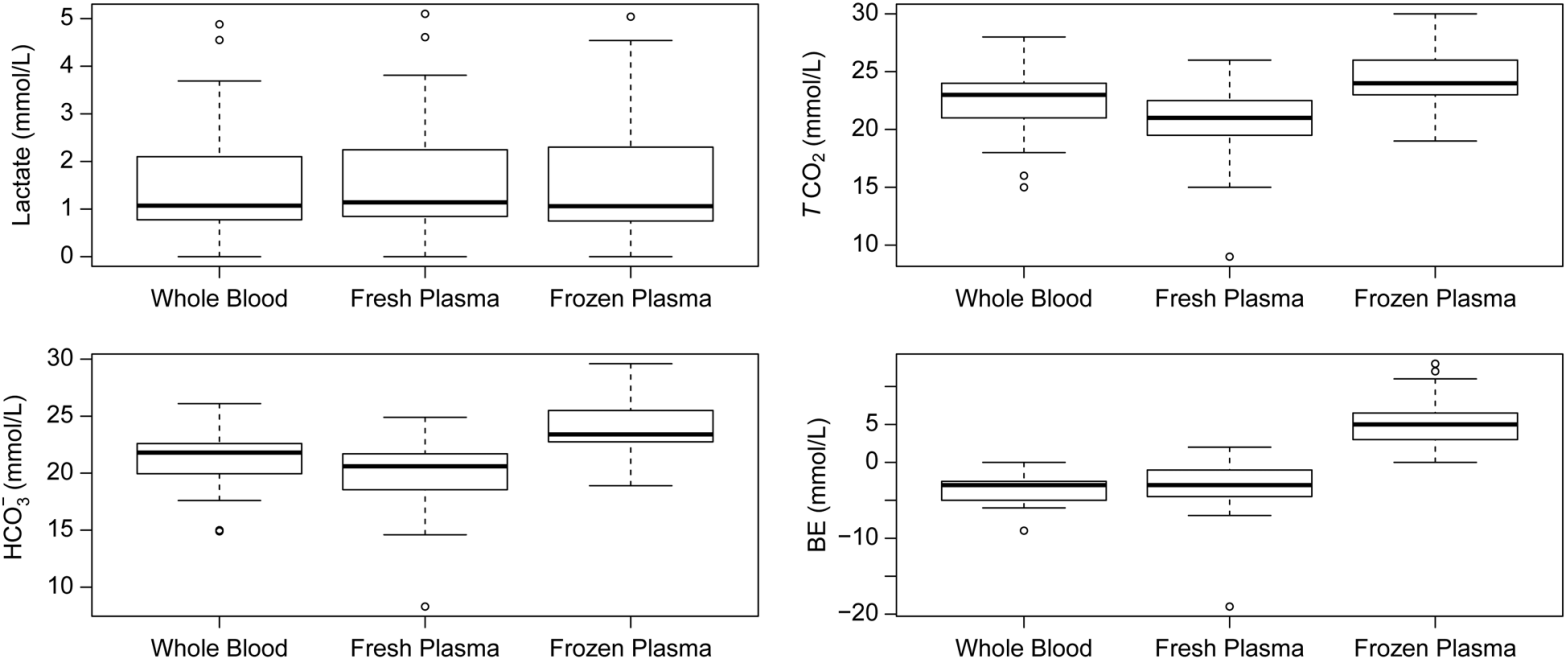
Box plots for lactate, total carbon dioxide (*T*CO_2_), bicarbonate (HCO_3_^−^) and base excess (BE) concentrations in American alligator (*Alligator mississippiensis*) whole blood, fresh plasma and frozen plasma samples.

We found that sample type had no effect on lactate concentration values (*F*_2,65_ = 0.37, *P* = 0.963; Fig. 1). However, sample type significantly affected pH (*F*_2,65_ = 100.00, *P* < 0.001), *P*CO_2_ (*F*_2,65_ = 59.97, *P* < 0.001), *P*O_2_ (*F*_2,65_ = 224.1, *P* < 0.001), concentration of HCO_3_^−^ (*F*_2,65_ = 10.97, *P* < 0.001), *T*CO_2_ (*F*_2,65_ = 9.707, *P* < 0.001), BE (*F*_2,65_ = 12.26, *P* < 0.001) and percentage sO_2_ (*F*_2,65_ = 31.8, *P* < 0.001; Table 1). *Post hoc* analysis indicated that pH and *P*O_2_ and *P*CO_2_ were significantly different among all three sample types (*P* ≤ 0.003). There was no difference between whole blood *T*CO_2_ and frozen (*P* = 0.079) or fresh plasma (*P* = 0.079), respectively (Fig. 1). However, *T*CO_2_ frozen plasma concentrations were significantly higher than fresh plasma samples (*P* < 0.001; Fig. 1). The HCO_3_^−^ and BE concentrations in frozen plasma were significantly higher than concentrations in both fresh plasma (*P* < 0.001 and *P* < 0.001, respectively) and whole blood (*P* = 0.018 and *P* < 0.001, respectively). However, there was no difference between whole blood and fresh plasma (HCO_3_^−^, *P* = 0.082; BE, *P* = 0.578; Fig. 1). Whole blood sO_2_ levels were significantly lower than both fresh plasma (*P* < 0.001) and frozen plasma (*P* < 0.001), but there was no difference between fresh plasma and frozen plasma samples (*P* = 0.19).

### Reference whole blood values

Blood samples from Session 1 (*n* = 8) were collected within 1 min of the animal being captured (seconds were not recorded). The mean sample collection time (±1 SD) during Session 2 (*n* = 15) was 46.2 ± 11.8 s. The mean time (±SD) from whole blood sample collection to complete sample analysis on the i-STAT was 4 ± 2.1 min. The majority of whole blood samples yielded complete biochemistry results (*n* = 19, 83%; Table 1). Whole blood concentrations of lactate from four individuals were below the detectable limit (<0.30 mmol/l; Table 1) of the analyser used for this study. None of the blood biochemistry analytes measured in whole blood was correlated with sample collection time.

## Discussion

### Effect of sample type on analytes

We found that sample type (i.e. whole blood, fresh plasma or frozen plasma) had no effect on lactate concentrations in American alligators, thus suggesting that both fresh and frozen (stored below −60°C) plasma samples can be evaluated reliably on the i-STAT analyser. It is important to note that lactate concentrations were relatively low in this study because of our efficient sampling protocols and that reptile lactate concentrations can increase rapidly in response to elevated activity (e.g. diving) and handling time (Coulson and Hernandez, 1979; Lewbart *et al.*, 2015). For example, alligator plasma lactate concentrations can exceed 30 mmol/l following short bursts of activity (Coulson and Hernandez, 1983), emphasizing the importance of capture and sampling techniques. In the present study, we investigated potential confounding variables associated with capture and sampling by completing a correlational analysis using lactate concentrations and sample collection time. Although we did not detect a correlation (*r*^2^ = 0.13, *P* = 0.087), it is important to emphasize that elevated blood and plasma lactate concentrations (>20 mmol/l), such as those associated with strenuous activity, may require dilution in order for lactate values to fall within the reportable range (Table 1) of an i-STAT analyser (Waldoch *et al.*, 2009; Abbott Point of Care Inc., 2013a). However, the suitability of diluting plasma samples for analysis on the i-STAT has yet to be investigated.

In contrast to lactate, fresh and frozen plasma sample pH, *P*CO_2_, *P*O_2_ and sO_2_ and frozen plasma concentrations of BE and HCO_3_^−^ varied greatly when compared with whole blood samples. Acid–base and blood gas values are susceptible to changes associated with prolonged sample exposure to aerobic conditions and the time between sample collection and analysis (Campbell, 2012). Furthermore, allowing blood to stand (with or without exposure to aerobic conditions) can influence the results of certain blood and plasma analytes (Burgdorf-Moisuk *et al.*, 2012; Abbott Point of Care Inc., 2013b). For example, exposure of a blood sample to non-anaerobic conditions allows CO_2_ to escape, which contributes to a decrease in *P*CO_2_ and increase in sample pH (Thrall *et al.*, 2012; Abbott Point of Care Inc., 2013c). Additionally, *T*CO_2_ concentrations are calculated from pH and *P*CO_2_ values, which are measured directly by the i-STAT using a standardized version of the Henderson–Hasselbalch equation (Abbott Point of Care Inc., 2013c). In the study by Ungerer *et al*. (1990), mean plasma sample *T*CO_2_ concentrations calculated by the Henerson–Hasselbalch equation were 0.71 mmol/l lower than those of whole blood samples even though anaerobic conditions were maintained during sample collection and processing. In our study, fresh plasma mean *T*CO_2_ values were 1.96 mmol/l lower than whole blood values (Table 1). However, mean frozen plasma sample *T*CO_2_ concentrations were 2.30 mmol/l higher than whole blood values (Table 1). Although we observed no significant differences between plasma or whole blood samples, we did observe a significant difference between the two plasma sample types (fresh vs. frozen), emphasizing the importance of pre-analytical sample handling procedures and the potential influence of sample type on analyte values.

In this study, we used whole blood sampling protocols similar to those used in other reptile POC blood gas and acid–base studies (Lewbart *et al.*, 2014, 2015). However, the necessary steps used for preparing a plasma sample for analysis in this study (i.e. inverting sample tubes to mix anticoagulant, centrifugation process) may have allowed for increased aerobic conditions and, consequently, influenced analyte results. Reducing sample centrifugation time and eliminating any sample mixing steps may mitigate any effects on blood gas concentrations and acid–base values in future studies. Furthermore, we attempted to standardize sample collection and handling among individual alligators and between sampling sessions to minimize unintended variation in analyte values. Using alternative sample collection and handling techniques, such as those described by Harter *et al.* (2015b), may reduce variation between individuals and increase the accuracy of analyte readings.

### Reference whole blood values

Although we are unable to provide formal reference intervals (*n* < 120 individuals; Geffré *et al.*, 2009) for American alligators in the present study, we are able to provide preliminary baseline values for all CG4+ cartridge analytes using fresh whole blood samples and for lactate using fresh and frozen plasma samples (Table 1). It is important to note that while POC devices are commonly incorporated in field studies focused on ectothermic species, the i-STAT was originally developed for use with humans in a clinical setting. Thus, the i-STAT analyses cartridges at a temperature of 37°C, and results are calculated based on blood attributes derived from human blood samples. Although the i-STAT does allow for temperature corrections, temperature discrepancies between human blood samples and a given species may contribute to inaccurate results caused by temperature changes incorporated by the POC analyser (Harter *et al.*, 2014; Malte *et al.*, 2014). Therefore, validation studies are important for understanding any differences between the values attained from a POC device and more traditional diagnostic equipment.

Non-mammalian validation studies have indicated that body temperature, nucleated red blood cells and an assortment of other blood characteristics may have the potential to influence analyte results and, subsequently, produce inaccurate values when compared with conventional analyses (Harter *et al.*, 2015a, b). However, species-specific conversion factors, such as those used by Gallagher *et al.* (2010), may provide a mechanism for converting values collected by an i-STAT analyser to those measured from traditional diagnostic equipment. Blood and plasma analyte values attained from any POC device not specified for a particular species should be approached with caution when compared with other forms of analysis until a thorough validation, such as those outlined by Harter *et al.* (2015a, b) and Stoot *et al.* (2014), has been completed. However, data reported in the present study do provide an important step in creating reference values for juvenile American alligators using this POC device.

### Conclusions and future studies

Field biologists and clinicians require species-specific reference values based on biochemical or haematological values of interest for POC devices to evaluate individual animal health or physiological status (Lewbart *et al.*, 2014). Lactate and blood gas concentrations in crocodilian arterial and venous blood have been used historically to evaluate digestion (Busk *et al.*, 2000), handling stress (Coulson and Hernandez, 1983; Franklin *et al.*, 2003; Olsson and Phalen, 2013), disease (La Grange and Mukaratirwa, 2014) and swimming performance (Gatten *et al.*, 1991). Evaluating the use of different sample types on a POC device, such as an i-STAT analyser, could provide researchers with the opportunity to use ‘banked’ samples and to include additional analyte analyses in future research. The results in the present study suggest that lactate can be measured reliably in American alligator whole blood and in both fresh and banked frozen plasma samples using the i-STAT analyser. Although the blood gas and acid–base values included in the CG4+ cartridge are susceptible to the effects of processing time, sample handling procedures and storage temperatures (Piccione *et al.*, 2007; Abbott Point of Care Inc., 2013d), our results provide support for using fresh and frozen plasma samples in an i-STAT device to determine multiple CG4+ cartridge analytes in American alligators. However, it is important to emphasize that the i-STAT was originally intended for use with mammals; therefore, caution must be employed when using the device in other non-mammalian species, and further validation is needed.

## Funding

This work was supported by the Department of Energy (award number DE-FC09-07SR22506) to the University of Georgia Research Foundation and by the Savannah River Nuclear Solutions – Area Completions Project.
